# Human emotion processing accuracy, negative biases, and fMRI activation are associated with childhood trauma

**DOI:** 10.3389/fpsyt.2023.1181785

**Published:** 2023-10-16

**Authors:** Alexis A. Reisch, Katie L. Bessette, Lisanne M. Jenkins, Kristy A. Skerrett, Laura B. Gabriel, Leah R. Kling, Jonathan P. Stange, Kelly A. Ryan, Mindy Westlund Schreiner, Sheila E. Crowell, Erin A. Kaufman, Scott A. Langenecker

**Affiliations:** ^1^Cognitive Neuroscience Center, Department of Psychiatry, University of Illinois at Chicago, Chicago, IL, United States; ^2^Department of Psychiatry and Huntsman Mental Health Institute, University of Utah, Salt Lake City, UT, United States; ^3^Semel Institute for Neuroscience and Human Behavior, University of California, Los Angeles, Los Angeles, CA, United States; ^4^Department of Psychiatry, University of Michigan, Ann Arbor, MI, United States; ^5^Departments of Psychology and Psychiatry and the Behavioral Sciences, University of Southern California, Los Angeles, CA, United States; ^6^Department of Psychology, University of Utah, Salt Lake City, UT, United States; ^7^Department of Obstetrics and Gynecology, University of Utah, Salt Lake City, UT, United States

**Keywords:** major depressive disorder, childhood trauma, facial emotion perception, response bias, middle frontal gyrus, facial emotion perception test

## Abstract

**Introduction:**

Emerging literature suggests that childhood trauma may influence facial emotion perception (FEP), with the potential to negatively bias both emotion perception and reactions to emotion-related inputs. Negative emotion perception biases are associated with a range of psychiatric and behavioral problems, potentially due or as a result of difficult social interactions. Unfortunately, there is a poor understanding of whether observed negative biases are related to childhood trauma history, depression history, or processes common to (and potentially causative of) both experiences.

**Methods:**

The present cross-sectional study examines the relation between FEP and neural activation during FEP with retrospectively reported childhood trauma in young adult participants with remitted major depressive disorder (rMDD, *n* = 41) and without psychiatric histories (healthy controls [HC], *n* = 34). Accuracy of emotion categorization and negative bias errors during FEP and brain activation were each measured during exposure to fearful, angry, happy, sad, and neutral faces. We examined participant behavioral and neural responses in relation to total reported severity of childhood abuse and neglect (assessed with the Childhood Trauma Questionnaire, CTQ).

**Results:**

Results corrected for multiple comparisons indicate that higher trauma scores were associated with greater likelihood of miscategorizing happy faces as angry. Activation in the right middle frontal gyrus (MFG) positively correlated with trauma scores when participants viewed faces that they correctly categorized as angry, fearful, sad, and happy.

**Discussion:**

Identifying the neural mechanisms by which childhood trauma and MDD may change facial emotion perception could inform targeted prevention efforts for MDD or related interpersonal difficulties.

## Introduction

Depression and childhood trauma are each related to difficulties in emotion perception, biases, as well as differences in functional activation and morphometric measurements of distributed brain regions including corticostriatal and corticolimbic circuits. In addition, childhood trauma is associated with increased risk of and specific trajectories for affective disorders [e.g., (1, 2)]. While such pathways of increased risk are well replicated in the literature, it is not yet clear what cognitive performance and brain developmental changes might drive such increased risk. We and others have proposed that changes in cognitive control might underlie such risk (3), but there are other (and many) possible risk factors. For example, commonalities in functional activation patterns are frequently reported in prefrontal and limbic regions (e.g., hippocampus, amygdala, dorsolateral prefrontal cortex [DLPFC], and anterior cingulate) and occasionally striatal regions [e.g., nucleus accumbens; (4)] during tasks that involve working memory and emotional conflict (5–11) It is possible that these observed activation differences in depression and childhood trauma relate to dysfunctional emotion processing (12, 13). For example, elevations in childhood trauma are correlated with altered performance on facial emotion perception (FEP) tasks, such that there is increased sensitivity to negative emotions, including a bias toward misperceiving negative emotions (12, 14–16). Notably, commonly reported elevated bilateral amygdala reactivity to sad faces in individuals exposed to childhood adversity [e.g., (17, 18)] is *not* associated with behavioral differences in accurately categorizing emotions (19), hinting that increased amygdala reactivity in these individuals is unrelated to FEP. Moreover, commonalities across depression and child trauma may be due to different experiences leading to similar outcomes (e.g., equifinality); for instance, both childhood trauma and parental warmth and support (even in the context of healthy development) impact amygdalar activation to negative faces in youth (20).

FEP difficulties are also, often observed among persons in both active and remitted states of major depressive disorder [MDD; (21)], and among groups at elevated risk for developing MDD (22–24), potentially through a stress sensitization process (25). Moreover, a consistent gaze bias to attend to sad faces is observed among individuals with MDD, irrespective of phase of illness (26, 27). Individuals with active MDD exhibit enhanced memory for previously displayed negative facial expressions, allocate more visual attention to sad faces than neutral faces (although sometimes only display less attention to happy faces (28), and are more likely to interpret emotionally neutral faces as sad compared with healthy controls [HCs; (12, 29, 30)]. Notably, individuals with remitted MDD (rMDD), who are at 4–6 times higher risk for experiencing another depressive episode, display an array of FEP biases, including greater facial fear recognition (31). Indeed, bias toward negative faces has predicted MDD relapse (32). Atypical patterns of responding to emotional inputs are also observed among youth at elevated risk of MDD. For example, children and adolescents of depressed mothers demonstrated greater attentional avoidance (gaze) of sad faces (33), and are more likely to categorize neutral stimuli as negative emotions (34, 35). Taken together, extant research suggests that individuals with active MDD, rMDD, and those at high risk of MDD each experience disruptions in FEP and related processes.

Even though emotion processing difficulties are associated with both childhood trauma and MDD, integrated efforts to understand these relationships and any dissociations between them are just beginning to be elucidated. Despite well-established links with trauma preceding negative affective biases and MDD, it still remains unclear whether negative affective biases or poor FEP accuracy are *driven* by trauma history, depression history, or processes common to both experiences. It is also unclear whether individuals’ histories are associated with unique neural patterns of responding during exposure to positive, negative, and neutral facial expressions. Of note, active symptoms of depression may obscure trait-based measurements of these cognitive processes [e.g., (36)]. For example, a bias to positive emotions (e.g., happy) could be present in remission, whereas a bias to negative emotions may be present during active high-symptom states. As the goal is to understand the effects of trauma rather than fluctuating symptom- related processes, this study recruited individuals in the remitted mood state. In fact, little is known about within-subject shifts in symptoms as they pertain to emotion processing biases.

The present study assesses FEP and negative biases via a forced-choice categorization paradigm of facial expressions among young adults with and without a history of MDD and varying levels of childhood trauma. It is designed to probe the effects of trauma history dimensionally among these two groups: individuals with and without MDD. In those with MDD, the potential confounding effects of active mood symptoms are reduced by examining individuals in the remitted phase. We hypothesized that (a) individuals with histories of more severe (retrospectively reported) childhood trauma would display a bias to categorize more faces incorrectly as fearful and angry, thus demonstrating a potentially adaptive response (a bias, but with lower accuracy overall) that may have been overgeneralized beyond the context of traumatic situations, and that (b) increased childhood trauma would have the greatest impact on the processing of fearful and angry faces in regions implicated in emotional reactivity and regulation (e.g., heightened amygdala activation and/or blunted DLPFC activation), which may be more pronounced among participants with rMDD.

## Methods

### Participants

Participants (ages 18 to 23) were young adults recruited with either no history of mental illness (healthy control, HC; *n* = 34), or with a history of MDD, currently remitted (rMDD; *n* = 41), as determined by qualified clinicians via clinical interview using diagnostic criteria from the DSM (Diagnostic Interview for Genetic Studies, or DIGS; (37). A parent or guardian completed a phone interview modified Family Interview for Genetic Studies) or medical records were obtained to confirm participant diagnoses and eligibility. Participants were recruited as part of a larger IRB approved study investigating neurobiological intermediate phenotypes in MDD. Procedures took place at two study sites: the University of Michigan (UM, 2011–2013) and then the University of Illinois at Chicago (UIC, 2013–2016).

Participants demographics are presented in Table 1. There were no exclusions based upon childhood trauma history. Exclusions were for active substance misuse; substance dependence (including alcohol and nicotine) within the past year; developmental disability (with the exception of attention-deficit/hyperactivity disorder); current active suicidal plan or intent; suicide attempt in the past six months; use of psychoactive medications within the past 30 days; symptoms of schizophrenia or psychosis; family history of schizophrenia or psychosis; and incompatibilities with MRI. Those classified in the HC group reported no prior psychiatric problems including a history of depressive episodes. Individuals with rMDD had relatively few prior depressive episodes (mode = 1 prior depressive episode), and had been in remission on average 3 years.

**Table 1:** Participant demographics.

	HC (*n* = 34)	rMDD (*n* = 41)
Mean age, years	20.67 (1.66)	21.39 (1.55)
Females, *n* (%)	21 (63.63)	26 (68.42)
Mean education, years (SD)	14.55 (1.42)	14.74 (1.35)
Mean IQ estimate (SD)^*^	106.00 (9.70)	107.10 (8.82)
Race, n (%)		
Black/African-American	1 (3%)	4 (10%)
Asian	5 (15%)	5 (12%)
Native American/Pacific Islander	0	0
Alaska Native	0	0
White	26 (76%)	28 (68%)
Multiracial/Mixed	0	2 (5%)
Hispanic, n (%)	4 (12%)	4 (10%)
Handedness (*n* right: *n* left) (*%* right)	31:3 (91%)	37:4 (90%)
Site, n (%)		
University of Michigan	11 (33%)	14 (37%)
University of Illinois at Chicago	22 (67%)	24 (63%)
*Illness characteristics*		
Past psychiatric medication, *n*^§^	NA	12 (31%)
History of co-morbid anxiety, *n*^§^	NA	13 (34%)
Number of MDEs (SD)^§^	NA	1.82 (1.20)
Age of first onset, years (SD)	NA	16.37 (3.35)
Age of last episode	NA	18.83 (3.86)
Current HDRS Recent Distress^*^	1.67 (1.12)	3.62 (1.72)
Beck anxiety inventory (SD)^*^	0.19 (1.02)	−0.20 (0.78)
Beck Depression Inventory (SD)^*^	1.90 (2.66)	4.40 (5.10)
FEPT accuracy	0.91 (1.53)	4.59 (6.20)
CTQ Total^^^^*^	81.10 (8.76)	80.56 (13.81)
Physical abuse	28.28 (4.15)	32.95 (9.01)
Emotional abuse^*^	5.53 (0.76)	5.78 (1.79)
Sexual abuse	6.00 (1.87)	8.03 (3.78)
Physical neglect	5.06 (0.25)	5.06 (0.35)
Emotional neglect	5.55 (1.05)	5.83 (1.50)
	6.40 (2.53)	7.92 (4.29)

CTQ, Childhood Trauma Questionnaire; HC, Healthy Controls; HDRS, Hamilton Depression Rating Score; IQ, Intelligence Quotient; MDE, Major Depressive Episodes; rMDD, remitted Major Depressive Disorder.

§ Exclusionary criteria for HCs.

^ Truncated 2 outliers.

* Group differences significant at *p* < 0.05.

### Measures

#### Childhood trauma questionnaire

The CTQ is a 28-item valid and reliable retrospective assessment that asks respondents to report on five subscales of abuse and neglect as experienced in their childhood. The CTQ demonstrates (38) strong psychometric properties in diverse clinical and community samples (39), and has shown high inter-rater kappa reliability in validation studies (40). The current study used the total score, which can range from 25 to 125, with a total clinical cut-off score of 49 (41). In the current sample, the total score ranged from 25 to 63 and reliability was good, α = 0.84.

#### Facial emotion perception test

**Figure 1:**
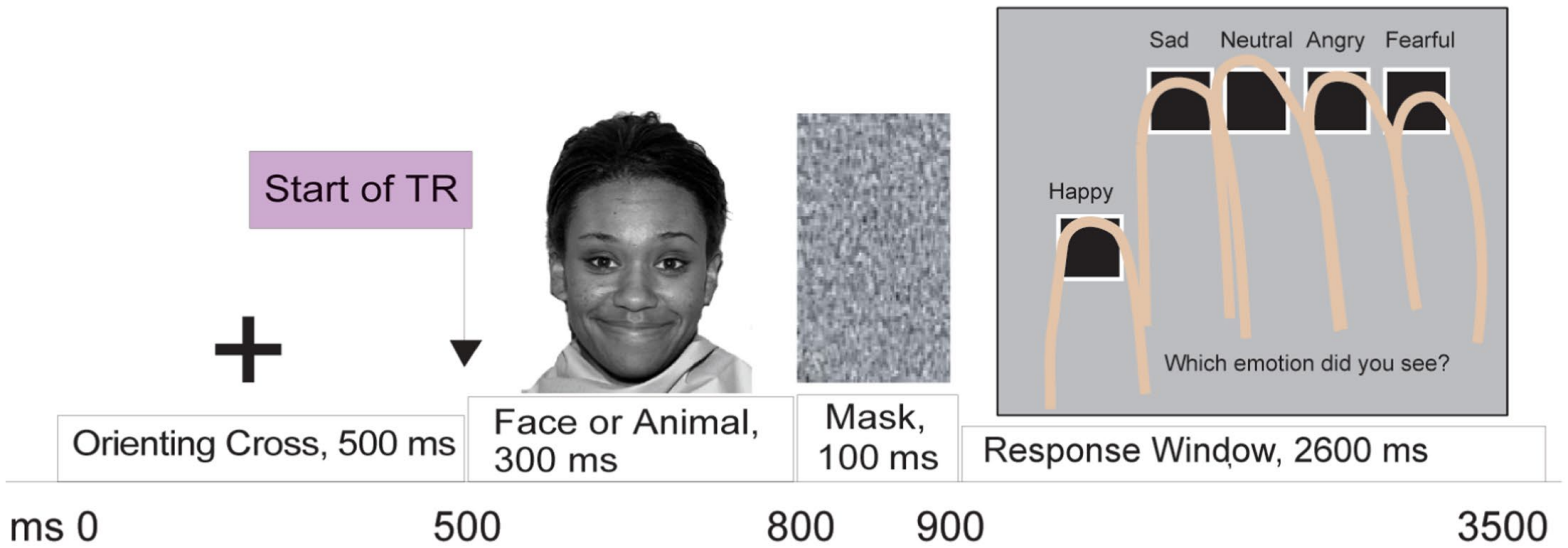
Visual depiction of sequence of stimuli displayed for a given trial in the Facial Emotion Perception Task (FEPT). Trials require participants either to categorize facial emotion depictions or broad animal types. Face reprinted with permission from The NimStim Set of Facial Expressions by Kapoor, A., Ewanation, L., Emamzadeh-Hashemi, E. A., Popov, M., Rashtbari, A., and Thölke, P., licensed under CC BY-4.0, retrieved from https://osf.io/y86rw/.

This task measures participants’ accuracy in categorizing facial expressions (21, 22, 42–44), and can be used to detect biases in facial emotion perception. The task includes 5 runs of the FEPT while in the MRI, with each run lasting 260 s and including 154 trials (see Figure 1). The FEPT has been described in detail elsewhere (21, 22) continues to use animals as a contrast, and uses the MacBrain Foundation Stimuli (45, 46).

#### Recent distress symptoms

A recent distress factor score was calculated (as a covariate of no interest) by performing a principal factor analysis (with regression factor) using the scores of the Beck Anxiety Inventory [BAI, (47) and the Beck Depression Inventory (BDI-II), (48, 49)]. These scales assess anxiety or depression symptoms in the past two weeks. These were combined because of a restriction of range due to selection criteria (i.e., in remission). Means for recent distress did not significantly differ between groups, as reported in Table 1.

### Procedures

In their first visit, participants completed informed consent in accordance with the Declaration of Helsinki, as well as diagnostic interviews and questionnaires. MRI procedures were completed during a separate second visit. On the second visit, participants were given full instructions and completed a practice run of the task that uses the Ekman and Friesen face stimuli (50). While in the scanner, participants completed five runs of this task each lasting 4 min and 20 s, for a total of 21 min and 20 s. As described in detail elsewhere (22), the FEPT task requires participants to choose between five categorical choices regarding a facial emotion expression or animal groups. The current study focuses entirely on the facial emotion perception component. Briefly, participants are shown a cross for 500 ms before a face appears for 300 ms displaying one of five emotions (happy, sad, angry, fearful, or neutral), quickly followed by a resampled grayscale mask for 100 ms. Participants are then given 2,600 ms to report which emotion was displayed, and are shown a screen indicating which finger corresponds to which emotion. The same button consistently represents the same emotion across all trials for each participant, but buttons are counterbalanced across participants. Participants are not given feedback post-response. The primary variables of interest for this study were the proportion of correctly identified emotional expressions for each emotion category. All participants received monetary compensation for their participation.

### Neuroimaging

Neuroimaging data were collected in a 3 Tesla scanner (Signa at UM or Discover at UIC, General Electric, Milwaukee, WI). For each run of the FEPT, blood-oxygen level dependent (BOLD) images were collected at UM in 29 interleaved 4 mm slices with a TR of 2 s, resulting in a total of 126 volumes collected per run, and at UIC in 44 interleaved 3 mm slices with a TR of 2 s. The task was presented using E-Prime 2.0 displayed on a monitor outside the scanner, and participants responded to the task using a five-button response ‘claw’ (51). Please see Appendix for additional details.

#### Processing of neuroimaging data

MRI data were preprocessed using SPM8 (https://www.fil.ion.ucl.ac.uk/spm/doc/) and AFNI (52). fMRI data were slice-time corrected and realigned to the 10th volume in FSL (http://fsl.fmrib.ox.ac.uk/fsl/fslwiki/) using MCFLIRT (53). Next, FSL’s Brain Extraction Tool was used to extract anatomical images, then SPM8 was used to co-register to functional images and normalize to Montreal Neurological Institute (MNI152) space. Smoothing was completed with a full width at half maximum kernal of 5 mm. First-level event-related models for each condition (fearful, angry, sad, happy, neutral correct responses) included deviations in translation and rotations along with first temporal derivative and square of parameters to estimate hemodynamic changes for each accurately categorized emotion aggregated over the multiple exposures (24–30 events for each emotion, only for correct responses).

Second-level brain activation models were created in SPM8 using a multiple regression model for each facial emotion category, with total CTQ score as the predictor of interest. To control for the potential effects of diagnosis and concurrent symptoms on both neurobiology and trauma recall, diagnosis, recent distress, and the number of days between clinical symptom measurement and MRI were included as covariates of no interest. In addition, sex, site, and average x, y, and z movement deviation in the scanner were added to the second-level model as covariates of no interest. A restricted space was also applied to the model using a dilated gray matter mask, so that only gray matter structures would be considered. Results are displayed on the average brain from the first 55 participants enrolled in the study. Whole brain analyses indicated a significant effect of activation for the relation of CTQ and all five facial emotions in right middle frontal gyrus (MFG).

#### *Post-hoc* ROI analyses

To follow-up this effect in right MFG, we examined associations with all emotions and during rest, using one 8 mm radius spherical ROI center (MNI coordinates *x*, *y*, *z* = +40, +41, +32) in the right middle frontal gyrus (MFG), extracted with MarsBaR. A partial correlation to compare the associations was then performed on the extracted values for rMFG and each emotion and resting blocks.

### Statistical analyses

Behavioral performance, demographic, and clinical data were analyzed using SPSS v26. There were two CTQ scores greater than three standard deviations from the mean, and these were truncated to three standard deviations from the mean (54). All FEPT variables demonstrated normality for skewness (±2) and kurtosis (±7), and all normality assumptions held. Pearson correlations were computed between accuracy of specific emotions and CTQ total score.

We also performed a partial correlation between CTQ total and the number of emotional face stimuli categorized in a certain way (e.g., number of neutral faces categorized as angry) while controlling for diagnosis (rMDD vs. HC), sex, site, and recent distress scores. Correlations were also run separately by diagnosis (HC and rMDD) to investigate specific effects of biases and childhood trauma within subgroups that might be distorted by known differences in emotion perception accuracy. Correlations that would survive false discovery rate (FDR) adjustment (55) for multiple comparisons (*q* = 0.25) are so noted.

## Results

### Performance and trauma results

The HC group demonstrated a significant inverse relation between CTQ scores and accurate identification of happy facial expressions (*r* = −0.54, *p* = 0.003), in addition to a positive relation between CTQ and no response to happy faces (*r* = 0.38, *p* = 0.04, Table 2). This effect was not present in the rMDD group. CTQ scores were not significantly related to FEP accuracy across groups for any of the other four emotion categories. In regards to errors, across the whole sample there was a positive association between CTQ total scores and a negative affective bias for categorizing happy faces as angry (whole sample: *r* = 0.40, *p* = 0.001; HC: *r* = 0.50, *p* = 0.006; rMDD: *r* = 0.32, *p* = 0.06). Among the HC group only, heightened CTQ scores were associated with miscategorizing happy faces as fearful (*r* = 0.40, *p* = 0.03) and miscategorizing neutral faces as angry (*r* = 0.39, *p* = 0.04), though these effects did not persist after FDR corrections.

**Table 2:** Peak MNI coordinates of brain regions with increased activation associated^§^ with greater CTQ, independent of other factors.

Lobe	Region	Emotion	BA	*x*	*y*	*z*	Voxels^*^ (*k*)	Peak Intensity (*Z*)
Frontal	Superior/Middle Frontal	Angry	9	38	42	34	51	3.2
		Fearful	9/10	44	40	28	71	3.27
		Happy	10	46	48	12	58	3.38
		Happy	9	28	44	38	128	3.53
		Neutral	9	38	34	42	10^**^	3.3
		Sad	9	40	40	32	15^**^	3.01
Limbic	Cingulate Gyrus	Fearful	31	14	−30	44	40	3.31
Occipital/Limbic	Lingual Gyrus/Posterior Cingulate	Angry	18/19	10	−54	2	177	4.14
Parietal	Inferior Parietal	Happy	40	38	−54	40	106	3.15

*Z*-values are based upon peak intensity at MNI coordinates listed.

§ No clusters were detected that show significantly lower activation with increasing CTQ at our voxel cutoff.

* Statistical threshold was set at a cutoff of k = 40 voxels and p = 0.005.

** For sad and neutral, cutoff was changed to k = 10 voxels to accommodate relevant cluster and for transparency and comparison.

### Neuroimaging results

#### Emotion-specific brain activation positively correlated with CTQ

**Figure 2:**
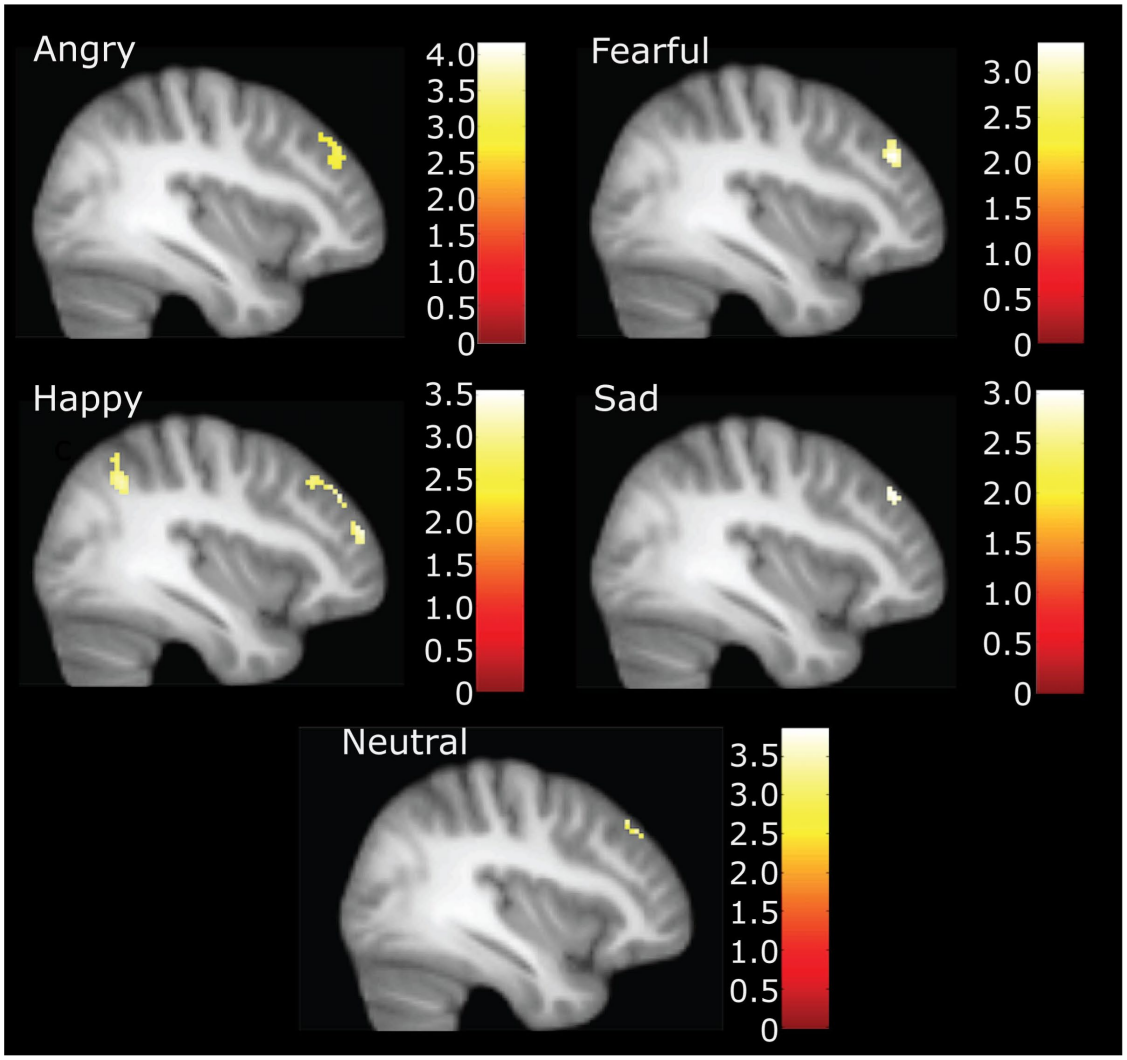
Neural activation during accurate categorization for angry, fearful, happy, sad and neutral emotions positively correlated with childhood trauma. Figure highlights right middle frontal gyrus activation at *X* = 38. Happy also demonstrates inferior parietal and other significant frontal clusters. Illustration only at *k* = 40 for angry, fearful, and happy. *For sad and neutral correlations, the voxel threshold was dropped to *k* = 10, *p* = 0.005 to display for comparison.

In the whole sample at the whole brain level, we observed higher CTQ total scores significantly associated with greater activation in the inferior parietal cortex (happy), posterior cingulate (angry) the cingulate gyrus (fearful), and the lingual (fearful) gyrus for distinct parts of emotional expression contrasts (see Table 2). No contrasts had activation that was inversely correlated with CTQ scores. The right middle frontal gyrus had a positive correlation of CTQ scores with activation for angry, fearful, sad, and happy, neutral face contrasts, in slightly different but largely overlapping areas (see Figure 2).

### MarsBaR ROI analysis for rMFG

**Figure 3:**
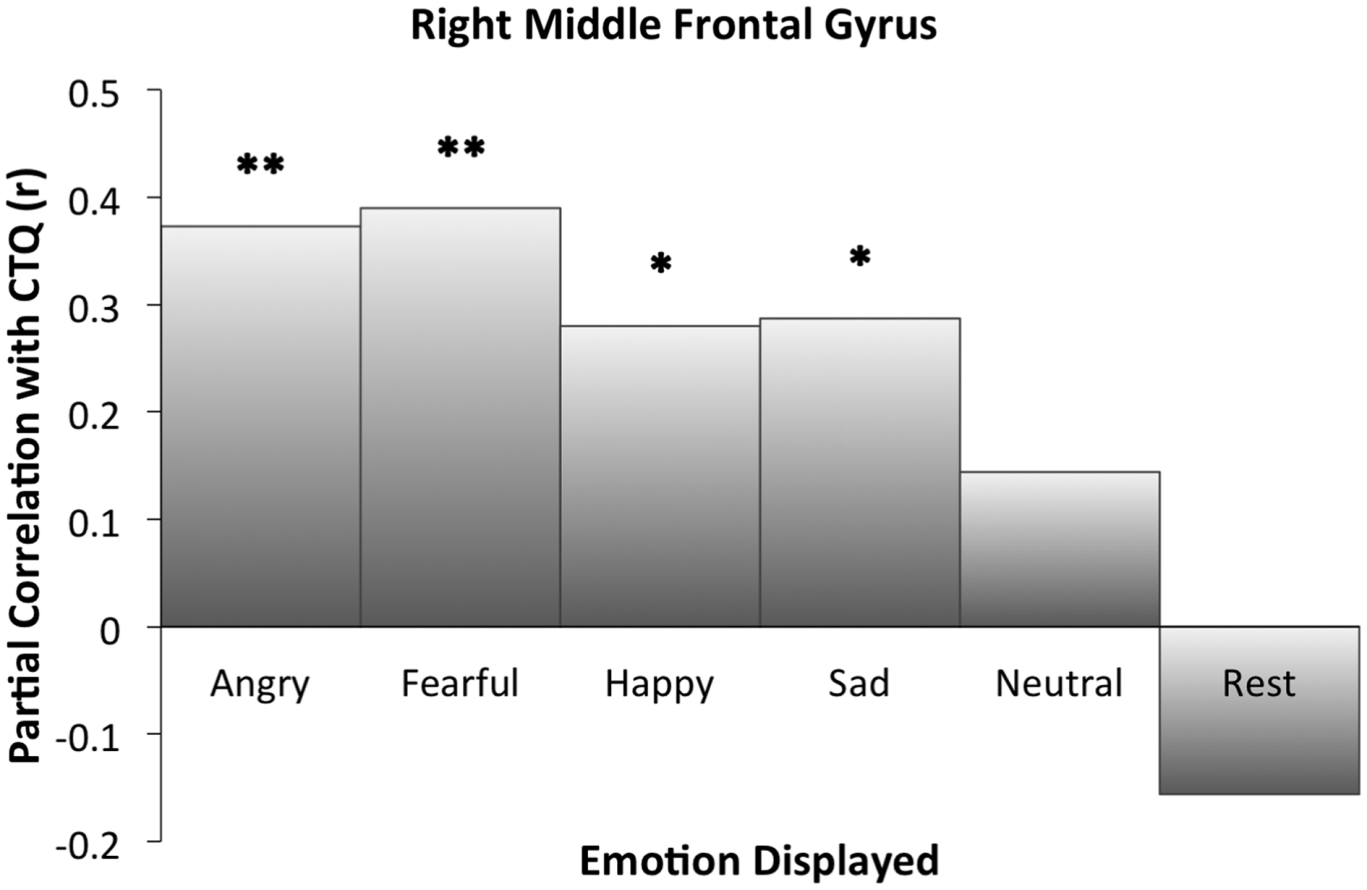
Right middle frontal gyrus activation partial correlations with childhood trauma (CTQ). Findings of the ROI analysis in an 8 mm cluster. Significant cluster activation differences occurred in relation to CTQ when correctly identifying angry, fearful, happy, and sad faces. For neutral and rest block conditions, CTQ was not significantly correlated with extracted activation within the cluster. *Indicates significant correlation between childhood trauma and activation for the specified condition. * *p* ≤ 0.05. ** *p* ≤ 0.01.

Right MFG cluster activity in all emotion conditions other than neutral was significantly correlated with CTQ scores, as displayed in Figure 2, with peak activation near *X* = 38 (see Table 3). Activation was positively correlated with CTQ scores for angry (*r* = 0.37, *p* < 0.01), fearful (*r* = 0.40, *p* < 0.01), happy (*r* = 0.30, *p* < 0.05), and sad (*r* = 0.29, *p* < 0.05) facial expressions. Figure 3 shows relations with other conditions (e.g., rest and neutral), which were not equivalent between HC and rMDD groups.

**Table 3:** Partial Correlations between accuracy and emotion biases on the FEPT with Total Childhood Trauma (CTQ).

	ALL (*df* = 65)	HC (*df* = 28)	rMDD (*df* = 33)
*Accuracy*			
Neutral	−0.04	0.03	−0.08
Happy	0.05	−0.54**^	0.15
Angry	0.11	0.11	0.16
Fearful	0.03	0.07	−0.01
Sad	0.03	−0.22	0.01
*Biases (Errors)*			
Neutral as fearful	−0.11	−0.30	−0.15
Neutral as angry	−0.02	0.39*	−0.17
Neutral as happy	0.16	0.27	0.13
Neutral as sad	0.03	0.14	0.01
Neutral no response	0.01	0.13	0.07
Happy as neutral	0.05	0.21	−0.27
Happy as fearful	−0.03	0.40*	−0.14
Happy as angry	0.40**^^^	0.50**^^^	0.32
Happy as sad	0.21	0.17	0.26
Happy no response	−0.09	0.38*	−0.13

2 CTQ total scores were truncated at 3 SD. df, degrees of freedom, df were adjusted after covariates.

*p ≤ 0.05, **p ≤ 0.01.

^ Benjamini–Hochberg significance with FDR *q* = 0.25.

## Discussion

The present study assessed FEP and negative affective bias via a forced-choice categorization paradigm of facial expressions among young adult participants with and without a history of MDD and varying levels of childhood trauma. We evaluated behavioral and neural response patterns for different facial emotions in relation to participants’ extent of past childhood trauma across HC and rMDD groups. This study expands upon previous emotion processing research by investigating the relations between childhood trauma and functional activation responses to social–emotional cues, independent of past depression history.

We found that individuals who reported higher levels of childhood trauma (regardless of diagnosis) demonstrated greater right MFG activity (part of DLPFC) during valenced (as opposed to neutral) emotional faces. We also observed across all participants that higher exposure to childhood trauma was related to miscategorization of happy faces as angry. Follow-up tests determined that HC participants with elevated childhood trauma tended to miscategorize neutral faces as angry more often than HC who reported less childhood trauma. Surprisingly, our whole-brain analyses did not reveal a relation between past childhood trauma and activation in the sgACC or the amygdala during exposure to emotional stimuli, but rather within right middle frontal gyrus (8, 9, 11).

Our finding of dimensional relations between trauma and FEP across diagnostic categories are consistent with our prior work examining inhibitory control and trauma in healthy individuals and those with bipolar disorder (56). Additional research is needed to extrapolate potential long-term influences that different types of trauma may have on emotion processing in adulthood, and how these processes may vary across persons with different mental health diagnoses. For example, only longitudinal studies like the ABCD can infer the sequential relations of trauma to brain changes in key processes that may be the foundation for risk for psychopathology.

Our hypothesis, that individuals with more severe childhood trauma would more frequently miscategorize faces as fearful and angry, was partially supported. We found that higher childhood trauma severity was associated with misperceiving happy faces as angry, regardless of diagnostic history. It is possible that abuse and neglect may lead persons to conflate others’ happiness and anger, because both emotions are associated with approach-related social behavior [e.g., (57)]. Prior research indicates that patterns of approach-avoidance behavior are associated with emotional valence evoked by environmental stimuli. Approach behaviors are typically linked to positive affect and can be associated with attack behaviors in the context of anger [e.g., (58)]. A further possibility is that a person engaging in abuse may appear happy while executing an angry response, which may cause the victim of abuse to link angry and happy emotions ⎯ particularly after repeated exposures (e.g., in cases where the person engaging in abuse is also a caregiver or loved one). The human brain is also primed to recognize fear-relevant or threat-inducing stimuli like anger more readily than other cues (59), and such a predisposition could be triggered by trauma experiences. In fact, one study found that humans detect angry faces more readily than happy faces among a crowd of people (60).

We hypothesized that childhood trauma would be most strongly associated with anger and fear processing as manifested by heightened amygdala and blunted DLPFC activation, and we did not observe either of these patterns. Amygdala activation appears to have prominent state-dependent (including with active depression) and individual differences effects that can be ascribed to processes other than excessive childhood trauma exposure or trait-based effects of MDD (61–65). Another surprising finding was that results yielded a *positive* rather than *negative* relation between activation within the MFG and childhood trauma. This opposite pattern may be due to the current sample’s *lack* of active mood symptoms, such that both blunted DLPFC activation and increased amygdala activation during FEP occurs only during an episode, with associated cognitive burden or loss of functioning. On the other hand, positive relations between activation in MFG and childhood trauma during accurate facial expression perception may demonstrate adaptive compensation or suppression of amygdala activation to emotionally salient stimuli. Indeed, Briceño et al. (66) found that those females with active MDD who shifted away from left DLPFC activation toward greater right DLPFC activation had greater emotion detection accuracy. We found that MFG activation was related to all emotionally valanced facial expressions as CTQ scores increased, rather than only fearful and angry faces. The presence of the same pattern, in both active and remitted MDD, could reflect a stable characteristic of trauma associated with brains. That is, activation within rMFG may be linked to how trauma history and emotionally salient stimuli are bound more generally, perhaps requiring more higher-order cognitive processing. While heightened MFG activation for emotional stimuli has been reported in those with active MDD (67–70), there is a paucity of studies of childhood trauma within the context of remitted MDD, Such studies are needed to dissociate whether activation patterns are related to trauma versus symptoms. It is possible that both childhood adversity and active depressive episodes result in elevated DLPFC activation and increased higher-order cognitive processing of emotional expressions, thereby a common pathway (67–70).

While neural results demonstrate group differences in brain activation and trauma histories on trials when participants *correctly* categorized the emotion of a facial expression, it is possible that interesting activation patterns related to childhood trauma may also present in the instances where participants *incorrectly* categorized a face. There was a surprising and interesting relationship where happy faces were more likely to be incorrectly labeled as angry in individuals with higher CTQ scores. Future studies could pursue this with longer versions of the test so that there would be enough errors to reliably model the BOLD signal for errors of different emotions.

A final consideration from this study is that neutral facial expression categorization and rest block conditions may not serve as adequate control condition(s) for FEP studies. Researchers often assume that neutral and rest block conditions capture “normative” activity that would be unrelated to trauma or disease. Many scientists use such designs to establish a baseline against which emotion evocation or FEP conditions can be compared to cancel out unrelated neural activation noise. Our results demonstrated that patterns of neural activation varied systematically with CTQ scores in *both* neutral facial expression trials and at rest (Figure 3). As such, using either neutral or rest block scans as a control condition (e.g., as part of a contrast between an emotion and neutral or rest) would have distorted and potentially hidden our current results with salient emotions. Moreover, our results suggest rest and neutral do not demonstrate activation equivalent relationships with childhood trauma that can be used interchangeably for creation of contrasts of interest. While a byproduct result of the current analyses, these results coalesce with others demonstrating significant differences in connectivity patterns at rest for individuals with childhood trauma [e.g., (71)]. Future studies would be best served by continuing to ensure equivalency in their control conditions across groups within their samples prior to examining contrast conditions.

Although this study design has several strengths, including investigation of childhood trauma in young adults while early in the course of recurrent MDD processes, limitations should be considered. First, we used a retrospective measure of childhood trauma, which may be influenced by environmental factors, symptoms, and poor recall, and therefore potentially limited in their validity (72, 73), although more recent studies suggest that these scores are stable over time in adulthood (74). We controlled for current symptoms through inclusion criteria (remission from depression) and inclusion of covariates diagnosis and recent distress in analyses, yet were unable to examine differential relations with adult trauma exposure. Prospective longitudinal studies, including adjustment for age of first trauma exposure, and measurement of adult traumas would more accurately probe relations between childhood trauma and emotion processing. Sample size and power to detect effects are long-standing difficulties in the field of neuroimaging; while the sample here was adequate to detect a main fixed effect of childhood trauma, the sample size was underpowered to detect emotion-related processing differences across diagnosis or trauma subscales. Analysis of specific types of abuse and neglect independently may reveal differential FEP and neurobiology, as each type is related to different outcomes [e.g., (75)]. A third limitation is that recent life traumas and stressors were not assessed. Future studies focused specifically on the developmental effects of childhood trauma should include a questionnaire to account for the potential influence of recent life stress and trauma occurring post-childhood. In future studies, scientists should recruit a larger sample of participants who have a wider range of childhood trauma severity.

## Conclusion

Childhood trauma is a multifaceted developmental experience. Researchers should continue to study distal effects of trauma on social emotional processing in order to hone prevention efforts and better elucidate risk for interpersonal difficulties and adverse health outcomes, including risk for MDD. The present study provides evidence that childhood trauma is related to emotion processing, categorization, and biased recognition accuracy as well as differing patterns of neural activation while engaged in emotion processing and categorization. Furthermore, some of these effects differed by individuals’ experiences of prior episodes of depression. Additional research is needed to extrapolate potential long-term influences that different types of trauma may have on emotion processing in adulthood, and how these processes may vary across persons with different mental health diagnoses.

## Data availability statement

The raw data supporting the conclusions of this article will be made available by the authors, without undue reservation.

## Ethics statement

The studies involving human participants were reviewed and approved by University of Michigan (UM) and the University of Illinois at Chicago (UIC). The patients/participants provided their written informed consent to participate in this study. Written informed consent was obtained from the individual(s) for the publication of any identifiable images or data included in this article.

## Author contributions

AR, KB, LJ, JS, KR, and SL: conceptualization. AR, KB, and SL: data curation. AR, KB, LJ, JS, and SL: formal analysis and writing – original draft. AR, EK, and SL: funding acquisition. AR, KS, LG, LK, and SL: investigation. AR, LG, LK, KR, MS, EK, SC, and SL: methodology. KB, KS, LG, LK, and SL: project administration. KR and SL: resources and software. KB, LG, LK, and SL: supervision. KB, MS, EK, SC, and SL: validation. KB, LJ, and SL: visualization. AR, KB, LJ, KS, LG, LK, JS, KR, MS, EK, SC, and SL: writing – review and editing. All authors contributed to the article and approved the submitted version.

## Appendix

### Exclusions from current analysis

133 participants were enrolled in the study, yet two were excluded from data collection due to symptoms of schizophrenia or schizotypal disorder. In addition following the collection of data, 60 participants who had participated in the study were excluded from the current analysis for the following reasons: not scanned or fMRI data was unusable (61), excessive head movement during FEPT (31), incomplete or missing questionnaires – study dropout (52), Hamilton Depression Rating (HDRS) score above 7 indicating active depression (40), and conversion to active depressed or bipolar during study timeline (47) software for analysis and visualization of functional magnetic).

### Scanner acquisition details

The UM scanner was a 3T Signa (release VH3, General Electric, USA) and used a reverse spiral sequence to acquire 36 slices 3.5 mm thick. The image matrix was 64 x 64 over a 22 cm field of view (FOV) for a 3.44 x 3.44 voxel. TR = 2000 ms, TE = 30 ms. Additionally, 116 high-resolution T1 spoiled gradient echo (SPGR) slices were collected for co-registration purposes, with slice thickness = 1.2 mm, field of view = 26cm, matrix size = 256 x 256.

The UIC scanner was a 3T GE Discovery and acquired images with a gradient-echo axial echo-planar imaging sequence. The image matrix was 64 x 64 over a 22 cm FOV with 3mm slice thickness (0 gap) for a 3.44 x 3.44 voxel. TR = 2000ms, TE = minFul (22.2ms), 90° flip, 44 slices (ascending, interleaved). The 4 initial scans were discarded, resulting in 126 volumes for each of five runs, each lasting 4 minutes 20 seconds. The anatomical scan was a T1SPGR echo, with a 22 cm FOV, 256 x 256 matrix size, 1 mm slice thickness, for a 0.86 x 0.86 voxel size, including 182 slices during a scan time of around 4 minutes.

## References

[1] Hoppen TH Chalder T. Childhood adversity as a transdiagnostic risk factor for affective disorders in adulthood: a systematic review focusing on biopsychosocial moderating and mediating variables. Clin Psychol Rev. (2018) 65:81–1. doi: 10.1016/j.cpr.2018.08.002, PMID: 30189342

[2] Watters ER Aloe AM Wojciak AS. Examining the associations between childhood trauma, resilience, and depression: a multivariate Meta-analysis. Trauma Violence Abuse. (2023) 24:231–4. doi: 10.1177/15248380211029397, PMID: 34313169

[3] Bessette KL Karstens AJ Crane NA Peters AT Stange JP Elverman KH. A lifespan model of interference resolution and inhibitory control: risk for depression and changes with illness progression. Neuropsychol Rev. (2003) 30:477–8. doi: 10.1007/s11065-019-09424-5, PMID: 31942706 PMC7363517

[4] Nagy SA Kurtos Z Nemeth N Perlaki G Csernela E Lakner FE. Childhood maltreatment results in altered deactivation of reward processing circuits in depressed patients: a functional magnetic resonance imaging study of a facial emotion recognition task. Neurobiol Stress. (2021) 15:100399. doi: 10.1016/j.ynstr.2021.100399, PMID: 34646916 PMC8495173

[5] Demers LA Hunt RH Cicchetti D Cohen-Gilbert JE Rogosch FA Toth SL. Impact of childhood maltreatment and resilience on behavioral and neural patterns of inhibitory control during emotional distraction. Dev Psychopathol. (2022) 34:1260–71. doi: 10.1017/S0954579421000055, PMID: 33827733 PMC8497637

[6] Demers LA Mckenzie KJ Hunt RH Cicchetti D Cowell RA Rogosch FA. Separable effects of childhood maltreatment and adult adaptive functioning on amygdala connectivity during emotion processing. Biol Psych Cogn Neurosci Neuroimaging. (2018) 3:116–4. doi: 10.1016/j.bpsc.2017.08.010, PMID: 29529406 PMC5851478

[7] Hart H Rubia K. Neuroimaging of child abuse: a critical review. Front Hum Neurosci. (2012) 6:52. doi: 10.3389/fnhum.2012.00052 22457645 PMC3307045

[8] Marusak HA Martin KR Etkin A Thomason ME. Childhood trauma exposure disrupts the automatic regulation of emotional processing. Neuropsychopharmacology. (2015) 40:1250–8. doi: 10.1038/npp.2014.311, PMID: 25413183 PMC4367470

[9] Philip NS Sweet LH Tyrka AR Carpenter SL Albright SE Price LH. Exposure to childhood trauma is associated with altered n-back activation and performance in healthy adults: implications for a commonly used working memory task. Brain Imaging Behav. (2016) 10:124–5. doi: 10.1007/s11682-015-9373-9, PMID: 25804310 PMC4583340

[10] Schulze L Schulze A Renneberg B Schmahl C Niedtfeld I. Neural correlates of affective disturbances: a comparative Meta-analysis of negative affect processing in borderline personality disorder, major depressive disorder, and posttraumatic stress disorder. Biol Psychiatry Cogn Neurosci Neuroimaging. (2019) 4:220–2. doi: 10.1016/j.bpsc.2018.11.004, PMID: 30581154

[11] Stuhrmann A Suslow T Dannlowski U. Facial emotion processing in major depression: a systematic review of neuroimaging findings. Biol Mood Anxiety Disord. (2011) 1:10. doi: 10.1186/2045-5380-1-10, PMID: 22738433 PMC3384264

[12] Antypa N Cerit H Kruijt AW Verhoeven FE Van Der Does AJ. Relationships among 5-HTT genotype, life events and gender in the recognition of facial emotions. Neuroscience. (2011) 172:303–3. doi: 10.1016/j.neuroscience.2010.10.042, PMID: 20971165

[13] Dargis M Newman J. Altered emotion modulated startle in women with a history of childhood neglect. Personal Individ Differ. (2016) 89:187–1. doi: 10.1016/j.paid.2015.10.014

[14] Nicol K Pope M Hall J. Facial emotion recognition in borderline personality: an association, with childhood experience. Psychiatry Res. (2014) 218:256–8. doi: 10.1016/j.psychres.2014.04.017, PMID: 24809243

[15] Russo M Mahon K Shanahan M Solon C Ramjas E Turpin J. The association between childhood trauma and facial emotion recognition in adults with bipolar disorder. Psychiatry Res. (2015) 229:771–6. doi: 10.1016/j.psychres.2015.08.004, PMID: 26272021 PMC4603568

[16] Young JC Widom CS. Long-term effects of child abuse and neglect on emotion processing in adulthood. Child Abuse Negl. (2014) 38:1369–81. doi: 10.1016/j.chiabu.2014.03.008, PMID: 24747007 PMC4117717

[17] Kessler R Schmitt S Sauder T Stein F Yuksel D Grotegerd D. Long-term neuroanatomical consequences of childhood maltreatment: reduced amygdala inhibition by medial prefrontal cortex. Front Syst Neurosci. (2020) 14:28. doi: 10.3389/fnsys.2020.00028, PMID: 32581732 PMC7283497

[18] Van Harmelen AL Van Tol MJ Demenescu LR Van Der Wee NJ Veltman DJ Aleman A. Enhanced amygdala reactivity to emotional faces in adults reporting childhood emotional maltreatment. Soc Cogn Affect Neurosci. (2013) 8:362–9. doi: 10.1093/scan/nss007, PMID: 22258799 PMC3624946

[19] Saarinen A Keltikangas-Jarvinen L Jaaskelainen E Huhtaniska S Pudas J Tovar-Perdomo S. Early adversity and emotion processing from faces: a meta-analysis on behavioral and neurophysiological responses. Biol Psychiatry Cogn Neurosci Neuroimaging. (2021) 6:692–5. doi: 10.1016/j.bpsc.2021.01.002, PMID: 33486133

[20] Romund L Raufelder D Flemming E Lorenz RC Pelz P Gleich T. Maternal parenting behavior and emotion processing in adolescents-an fMRI study. Biol Psychol. (2016) 120:120–5. doi: 10.1016/j.biopsycho.2016.09.003, PMID: 27645501

[21] Langenecker SA Bieliauskas LA Rapport LJ Zubieta JK Wilde EA Berent S. Face emotion perception and executive functioning deficits in depression. J Clin Exp Neuropsychol. (2005) 27:320–3. doi: 10.1080/13803390490490515720 15969355

[22] Jenkins L Kassel M Gabriel L Gowins JR Hymen E Verges A. Amygdala and dorsomedial activity during facial emotion processing in youth with remitted major depression. Soc Cogn Affect Neurosci. (2016) 11:736–5. doi: 10.1093/scan/nsv152 26714574 PMC4847691

[23] Kohler CG Hoffman LJ Eastman LB Healey K Moberg PJ. Facial emotion perception in depression and bipolar disorder: a quantitative review. Psychiatry Res. (2011) 188:303–9. doi: 10.1016/j.psychres.2011.04.019 21601927

[24] Langenecker SA Jacobs RH Passarotti AM. Current neural and behavioral dimensional constructs across mood disorders. Curr Behav Neurosci Rep. (2014) 1:144–3. doi: 10.1007/s40473-014-0018-x, PMID: 25147755 PMC4136393

[25] Brown A Bennet J Rapee RM Hirshfeld-Becker DR Bayer JK. Exploring the stress sensitization theory with temperamentally inhibited children: a population-based study. BMC Pediatr. (2020) 20:264. doi: 10.1186/s12887-020-02159-w, PMID: 32471371 PMC7260781

[26] Bouhuys AL Geerts E Mersch PP Jenner JA. Nonverbal interpersonal sensitivity and persistence of depression: perception of emotions in schematic faces. Psychiatry Res. (1999) 64:193–3. doi: 10.1016/S0165-1781(96)02930-7, PMID: 8944397

[27] Joormann J Gotlib IH. Selective attention to emotional faces following recovery from depression. J Abnorm Psychol. (2007) 116:80–5. doi: 10.1037/0021-843X.116.1.80, PMID: 17324018

[28] Bodenschatz CM Skopinceva M Russ T Suslow T. Attentional bias and childhood maltreatment in clinical depression - an eye-tracking study. J Psychiatr Res. (2019) 112:83–8. doi: 10.1016/j.jpsychires.2019.02.025, PMID: 30870713

[29] Dalili MN Penton-Voak IS Harmer CJ Munafo MR. Meta-analysis of emotion recognition deficits in major depressive disorder. Psychol Med. (2015) 45:1135–44. doi: 10.1017/S0033291714002591, PMID: 25395075 PMC4712476

[30] Leppanen JM. Emotional information processing in mood disorders: a review of behavioral and neuroimaging findings. Curr Opin Psychiatry. (2006) 19:34–9. doi: 10.1097/01.yco.0000191500.46411.00 16612176

[31] Bhagwagar Z Cowen PJ Goodwin GM Harmer CJ. Normalization of enhanced fear recognition by acute SSRI treatment in subjects with a previous history of depression. Am J Psychiatry. (2003) 161:166–8. doi: 10.1176/appi.ajp.161.1.166, PMID: 14702268

[32] Bouhuys AL Geerts E Gordijn MCM. Depressed patients' perceptions of facial emotions in depressed and remitted states are associated with relapse: a longitudinal study. J Nerv Ment Dis. (1999) 187:595–2. doi: 10.1097/00005053-199910000-00002, PMID: 10535652

[33] Gibb BE Benas JS Grassia M Mcgeary J. Children's attentional biases and 5-HTTLPR genotype: potential mechanisms linking mother and child depression. J Clin Child Adolesc Psychol. (2009) 38:415–6. doi: 10.1080/15374410902851705, PMID: 19437301 PMC4113083

[34] Dearing KF Gotlib IH. Interpretation of ambiguous information in girls at risk for depression. J Abnorm Child Psychol. (2009) 37:79–91. doi: 10.1007/s10802-008-9259-z, PMID: 18679791 PMC2836928

[35] Platt B Cohen Kadosh K Lau JY. The role of peer rejection in adolescent depression. Depress Anxiety. (2013) 30:809–1. doi: 10.1002/da.22120, PMID: 23596129

[36] Cerny BM Stange JP Kling LR Hamlat EJ O'donnell LA Deveney C. Self-reported affective biases, but not all affective performance biases, are present in depression remission. Br J Clin Psychol. (2019) 58:274–8. doi: 10.1111/bjc.12217, PMID: 30854675 PMC6682436

[37] Nurnberger JI Blehar MC Kaufmann CA York-Cooler C Simpson SG Harkavy-Friedman J. Diagnostic interview for genetic studies. Rationale, unique features, and training. NIMH Genet Initiat ArchGenPsychiatry. (1994) 51:849–9. doi: 10.1001/archpsyc.1994.03950110009002 7944874

[38] Bernstein DP Ahluvalia T Pogge D Handelsman L. Validity of the childhood trauma questionnaire in an adolescent psychiatric population. J Am Acad Child Adolesc Psychiatry. (1997) 36:340–8. doi: 10.1097/00004583-199703000-00012 9055514

[39] Scher CD Stein MB Asmundson GJ Mccreary DR Forde DR. The childhood trauma questionnaire in a community sample: psychometric properties and normative data. J Trauma Stress. (2001) 14:843–7. doi: 10.1023/A:1013058625719, PMID: 11776429

[40] Bernstein DP Stein JA Newcomb MD Walker E Pogge D Ahluvalia T. Childhood trauma questionnaire--short form. Child Abuse Negl. (2003) 27:169–10. PMID: 12615092 10.1016/s0145-2134(02)00541-0

[41] Walker EA Gelfand A Katon WJ Koss MP Von Korff M Bernstein D. Adult health status of women with histories of childhood abuse and neglect. Am J Med. 107:332–9. doi: 10.1016/S0002-9343(99)00235-1 10527034

[42] Briceno EM Rapport LJ Kassel MT Bieliauskas LA Zubieta JK Weisenbach SL. Age and gender modulate the neural circuitry supporting facial emotion processing in adults with major depressive disorder. Am J Geriatr Psychiatry. (2015) 23:304–3. doi: 10.1016/j.jagp.2014.05.007, PMID: 25085721 PMC4241383

[43] Langenecker SA Caveney AF Giordani B Young EA Nielson KA Rapport LJ. The sensitivity and psychometric properties of a brief computer-based cognitive screening battery in a depression clinic. Psychiatry Res. (2007) 152:143–4. doi: 10.1016/j.psychres.2006.03.019, PMID: 17445911

[44] Weisenbach SL Rapport LJ Briceno EM Haase BD Vederman AC. Reduced emotion processing efficiency in healthy males relative to females. Soc Cogn Affect Neurosci. (2014) 9:316–5. doi: 10.1093/scan/nss137, PMID: 23196633 PMC3980801

[45] Jenkins LM Kendall AD Kassel MT Patron VG Gowins JR Dion C. Considering sex differences clarifies the effects of depression on facial emotion processing during fMRI. J Affect Disord. (2018) 225:129–6. doi: 10.1016/j.jad.2017.08.027, PMID: 28826089 PMC5626645

[46] Tottenham N Tanaka JW Leon AC Mccarry T Nurse M Hare TA. The NimStim set of facial expressions: judgments from untrained research participants. Psychiatry Res. (2009) 168:242–9. doi: 10.1016/j.psychres.2008.05.006, PMID: 19564050 PMC3474329

[47] Beck AT Epstein N Brown G Steer RA. An inventory for measuring clinical anxiety: psychometric properties. J Consult Clin Psychol. (1988) 56:893–7. doi: 10.1037/0022-006X.56.6.893 3204199

[48] Beck AT Steer RA Brown GK. Beck depression inventory-II. J San Antonio. (1996a) 78:490–8.

[49] Beck AT Steer RA Brown GK. Manual for the Beck depression inventory - II. Agra: Psychological Corporation (1996b).

[50] Ekman P Friesen. Pictures of facial affect. Palo Alto, CA: C.P. Press (1976).

[51] Psychology Software Tools, I. (2007). E-Prime 2.0. Pittsburgh, PA.

[52] Cox RW. AFNI: software for analysis and visualization of functional magnetic resonance neuroimages. Comput Biomed Res. (1996) 29:162–3. doi: 10.1006/cbmr.1996.0014, PMID: 8812068

[53] Jenkinson M Bannister P Brady M Smith S. Improved optimization for the robust and accurate linear registration and motion correction of brain images. NeuroImage. (2002) 17:825–1. doi: 10.1006/nimg.2002.1132, PMID: 12377157

[54] Tabachnick B Fidell L. Using multivariate statistics. Boston, MA: Pearson Education (2007).

[55] Benjamini Y Hochberg Y. Controlling the false discovery rate: a practical and powerful approach to multiple testing. J R Statis Soc Ser B Methodol. (1995) 57:289–10. doi: 10.1111/j.2517-6161.1995.tb02031.x

[56] Marshall DF Passarotti AM Ryan KA Kamali M Saunders EFH Pester B. Deficient inhibitory control as an outcome of childhood trauma. Psychiatry Res. (2016) 235:7–12. doi: 10.1016/j.psychres.2015.12.013, PMID: 26707783 PMC6639093

[57] Harmon-Jones C Schmeichel BJ Mennitt E Harmon-Jones E. The expression of determination: similarities between anger and approach-related positive affect. J Pers Soc Psychol. (2011) 100:172–1. doi: 10.1037/a0020966, PMID: 20853981

[58] Lang PJ Bradley MM. Emotion and the motivational brain. Biol Psychol. (2010) 84:437–10. doi: 10.1016/j.biopsycho.2009.10.007, PMID: 19879918 PMC3612949

[59] Fox E Lester V Russo R Bowles RJ Pichler A Dutton K. Facial expressions of emotion: are angry faces detected more efficiently? Cogn Emot. (2000) 14:61–92. doi: 10.1080/026999300378996, PMID: 17401453 PMC1839771

[60] Hansen CH Hansen RD. Finding the face in the crowd: an anger superiority effect. J Pers Soc Psychol. (1988) 54:917–4. doi: 10.1037/0022-3514.54.6.917 3397866

[61] Gerber AJ Posner J Gorman D Colibazzi T Yu S Wang Z. An affective circumplex model of neural systems subserving valence, arousal, and cognitive overlay during the appraisal of emotional faces. Neuropsychologia. (2008) 46:2129–39. doi: 10.1016/j.neuropsychologia.2008.02.032, PMID: 18440572 PMC2486369

[62] Olsavsky AK Brotman MA Rutenberg JG Muhrer EJ Deveney CM Fromm SJ. Amygdala hyperactivation during face emotion processing in unaffected youth at risk for bipolar disorder. J Am Acad Child Adolesc Psychiatry. (2012) 51:294–3. doi: 10.1016/j.jaac.2011.12.008, PMID: 22365465 PMC3292775

[63] Phan KL Fitzgerald DA Nathan PJ Tancer ME. Association between amygdala hyperactivity to harsh faces and severity of social anxiety in generalized social phobia. Biol Psychiatry. (2006) 59:424–9. doi: 10.1016/j.biopsych.2005.08.012, PMID: 16256956

[64] Taylor SF Kang J Brege IS Tso IF Hosanagar A Johnson TD. Meta-analysis of functional neuroimaging studies of emotion perception and experience in schizophrenia. Biol Psychiatry. (2012) 71:136–5. doi: 10.1016/j.biopsych.2011.09.007, PMID: 21993193 PMC3237865

[65] Weldon AL Hagan M Van Meter A Jacobs RH Kassel M Hazlett KE. Stress response to the functional magnetic resonance imaging environment in healthy adults relates to the degree of limbic reactivity during emotion processing. Neuropsychobiology. (2015) 71:85–96. doi: 10.1159/000369027, PMID: 25871424 PMC6679601

[66] Briceno EM Weisenbach SL Rapport LJ Hazlett EA Bieliauskas LA Haase BD. Shifted laterality in inferior frontal activation during emotion processing in women with major depressive disorder. Psychol Med. (2013) 10.1017/S0033291712002176 PMC4380502 23298715

[67] Lawrence NS Williams AM Surguladze S Giampietro V Brammer MJ Andrew C. Subcortical and ventral prefrontal cortical neural responses to facial expressions distinguish patients with bipolar disorder and major depression. Biol Psychiatry. (2004) 55:578–7. doi: 10.1016/j.biopsych.2003.11.017, PMID: 15013826

[68] Sheline YI Barch DM Donnelly J Ollinger J Snyder AZ Mintun MA. Increased amygdala response to masked emotional faces in depressed subjects resolves with antidepressant treatment: an fMRI study. Biol Psychiatry. (2001) 50:651–8. doi: 10.1016/S0006-3223(01)01263-X, PMID: 11704071

[69] Struyker Boudier H Teppema L Cools A Van Rossum J. (3,4-Dihydroxy-phenylamino)-2-imidazoline (DPI), a new potent agonist at dopamine receptors mediating neuronal inhibition. J Pharm Pharmacol. (1975) 27:882–3. doi: 10.1111/j.2042-7158.1975.tb10240.x, PMID: 1506

[70] Surguladze SA Young AW Senior C Brebion G Travis MJ Phillips ML. Recognition accuracy and response bias to happy and sad facial expressions in patients with major depression. Neuropsychology. (2004) 18:212–8. doi: 10.1037/0894-4105.18.2.212, PMID: 15099143

[71] Quinn ME Stange JP Jenkins LM Corwin S Deldonno SR Bessette KL. Cognitive control and network disruption in remitted depression: a correlate of childhood adversity. Soc Cogn Affect Neurosci. (2018) 13:1081–90. doi: 10.1093/scan/nsy077, PMID: 30285231 PMC6204481

[72] Hardt J Rutter M. Validity of adult retrospective reports of adverse childhood experiences: review of the evidence. J Child Psychol Psychiatry. (2004) 45:260–3. doi: 10.1111/j.1469-7610.2004.00218.x, PMID: 14982240

[73] Widom CS Raphael KG Dumont KA. The case for prospective longitudinal studies in child maltreatment research: commentary on Dube, Williamson, Thompson, Felitti, and Anda (2004). Child Abuse Negl. (2004) 28:715–2. doi: 10.1016/j.chiabu.2004.03.009 15261466

[74] Wang X Ding F Cheng C He J Wang X Yao S. Psychometric properties and measurement invariance of the childhood trauma questionnaire (short form) across genders, time points and presence of major depressive disorder among Chinese adolescents. Front Psychol. (2022) 13:816051. doi: 10.3389/fpsyg.2022.816051, PMID: 35478747 PMC9036057

[75] Hildyard KL Wolfe DA. Child neglect: developmental issues and outcomes. Child Abuse Negl. (2002) 26:679–5. doi: 10.1016/S0145-2134(02)00341-1 12201162

